# Solvent Influence on Absorption Spectra and Tautomeric Equilibria of Symmetric Azomethine‐Functionalized Derivatives: Structural Elucidation and Computational Studies

**DOI:** 10.1002/open.202100237

**Published:** 2022-02-22

**Authors:** Kifah S. M. Salih

**Affiliations:** ^1^ Department of Chemistry and Earth Sciences College of Arts and Sciences Qatar University P. O. Box 2713 Doha State of Qatar

**Keywords:** azomethine, solvatochromism, surface analysis, tautomers, Tauc plot

## Abstract

A new series of azomethine‐functionalized compounds was synthesized from the condensation of 2‐hydroxy‐1,3‐propanediamine and 2‐thienylcarboxaldehydes in the presence of a drying agent. The derivatives were spectroscopically characterized by NMR, LC‐MS, UV/Vis, IR and elemental analysis. Variable temperature ^1^H‐NMR (−60 to +60 °C) was performed to investigate the effect of solvent polarity; the capability of solvent to form H‐bond was found to dramatically influencing the tautomerization process of the desired structures. The calculated thermochemical parameters (ΔH_298_, ΔG_298_ and ΔS_298_) at DFT and MP2 levels of theory explained that **3 b** exists in equilibrium with two tautomers. The basis of the electronic absorptions was pursued through Time‐Dependent Density‐Functional Theory (TD‐DFT). Analysis of the structural surfaces was inspected and the molecular electrostatic potential (MEP) demonstrated that the three functionalized compounds were relatively analogous in the electronic distributions. Furthermore, the electrophilic and nucleophilic centers lying on the molecular surfaces were probably playing a key‐role in stabilizing the compounds through the nonclassical C−H⋅⋅⋅π interactions and hydrogen bonding. The impact of solvent polarity on absorption spectra were investigated via solvatochromic shifts. For instance, compound **3 c** displayed a gradual shift of the maximum absorption to the red area when the solvent polarity was increased, recording a 21 nm of bathochromic shift. In contrast, no significant solvent‐effect on **3 a** and **3 b** was observed. The solvation relation was pursued between Gutmann's donicity numbers the experimental λ_max_; exhibited almost positive linear performance with a minor oscillation, that ascribe to the possible weak interface between the molecules of solute and designated solvents. The bandgap energy of all products were assessed experimentally using optical absorption spectra following Tauc approach, giving −4.050 (**3 a**), −3.900 (**3 b**) and −3.210 (**3 c**) eV. However, the ΔE were computationally figured out from TD‐DFT simulation to be −4.258 (**3 a**), −4.022 (**3 b**) and −3.390 (**3 c**) eV.

## Graphical abstract

**The tautomerization of a series** of azomethine‐functionalized compounds is strongly influenced by the type of solvent used. An increase in solvent polarity results in a gradual shift of the maximum absorption. The bandgap energy of all products was assessed computationally by DFT and experimentally using optical spectra following the Tauc approach.

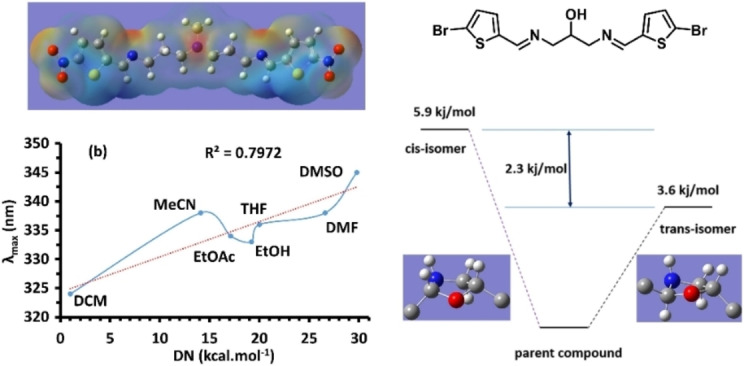

## Introduction

The design and synthesis of multi‐functionalized organic compounds for various applications are one of the focus of researchers and scientists in field of synthetic chemistry. Azomethine‐containing compounds display a fundamental role in tuning structure and altering physicochemical properties. These functional groups are predominantly recognized as Schiff bases or imine groups and present in numerous synthesized as well as natural‐occurring structures. Since this substantial moiety has been receiving significant considerations, various studies have exposed the applications and impacts of structural variation in fields of chemistry and biology. Azomethine compounds exhibits effective biological activities, such as anti‐inflammatory, antibacterial, antimalarial, antiviral, antiproliferative, antifungal, and antipyretic properties. Azomethine derivatives have been further employed as dyes and pigments, polymer stabilizers and as intermediates in synthetic fields.^[1]^

**Figure 1:**
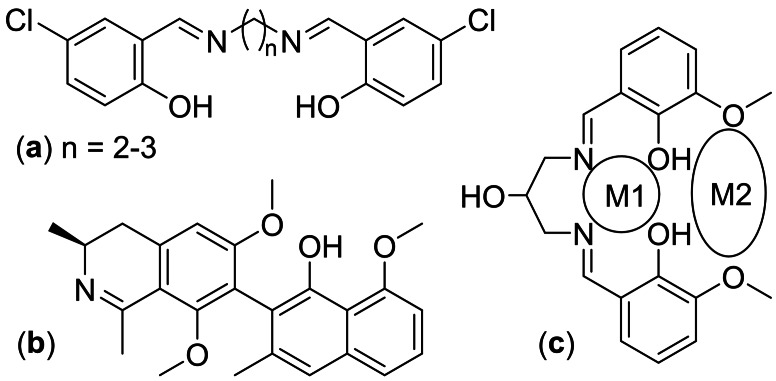
Antibacterial chloro‐salicylaldehyde‐SB derivatives (a) plant‐based antimalarial ancistrocladidine; (b) and a multidentate ligand for heterodinuclear architectures (c).

Azomethine derivatives represent as a crucial point in progressing complexation chemistry. The great tendency for coordination with transition metal ions allows this attractive group to turn as sensors and additionally took a specific role in the advancement of bioinorganic chemistry and manufacturing of optical materials.^[2]^ For example, azomethine‐containing compounds resulting from the dehydration of symmetric diamines or diamino alcohols with salicylaldehyde derivatives were accounted as potentially biscompartmental multidentate ligands with different electronic and steric properties. Such compounds demonstrated great affinity towards the formation of supramolecular architectures and coordination with different metal ions of s‐, p‐, d‐ and f‐block.^[3]^ Figure 1 demonstrates a number of azomethine‐functionalized compounds with significant applications.

The structural diversity of the resulting multinuclear transition metal complexes is prevailingly stabilized by various forces and interactions. Thus, the interest for such precursors has been increased predominantly in the field of crystal engineering and in electric/magnetic properties.^[4]^

Owing to their straightforward synthesis, satisfactorily isolation, efficient physicochemical properties, and their broad applicability, azomethine compounds have a great potential as functional group. For their electronic and geometric characteristics, these derivatives allow a smooth engagement with the host precursor, putting on notable selectivity and stability for the host‐guest complexation. Moreover, the participation in building H‐bond through the N atom with external or internal OH or NH functional moieties would tolerate the formation of robust structure. As non‐covalent interactions are included in the electronic perturbation inside the molecular bonded systems, they are indispensable to grant considerable molecular stability in solid and liquid states, though they are infirm in respect to their covalent counterparts.^[5]^

When one of the structural isomers is resulting from relocation of a proton and presents in very low concentration, they are regularly described as tautomers.^[6]^ Tautomers are constitutional isomers of chemical compounds that spontaneously interconvert into each other in chemical equilibrium with a low energy barrier. Fundamentally, there are two distinct sorts of tautomerism that could be linked to an intramolecular ring opening or closing process. The first, valence tautomerism, is described as fast reshuffling of the molecule's bonding electrons chaperoned by a geometric rearrangement. While the second encompasses of shifting a proton followed by a fluctuation in the molecule's bonding, known as prototropic tautomerism.^[7]^ The latter is the most popular type of tautomerism, a typical example includes the switching between keto and enol forms. However, ring‐chain tautomerism includes the relocation of H atom and subsequently followed by ring opening or closing, which often takes place in sugars, such as glucose. The ambient conditions, such as type of solvent, temperature, pressure and pH play a crucial role in the equilibrium between tautomers.^[8]^

In this article, the synthesis of novel products of 1,3‐bis(((*E*)‐(thiophen‐2‐yl)methylene)amino)propan‐2‐ol from typical condensation of 2‐hydroxy‐1,3‐propanediamine and 2‐thienylcarboxaldehydes is described. Full identification of the target compounds were performed using FT‐IR, ^1^H‐ and ^13^C‐NMR, UV/Vis, LC‐MS, and CHN analyses. Variable temperature ^1^H‐NMR was implemented to investigate the possible tautomeric structures. Surface analysis, optical behavior and the impact of solvent on tautomeric equilibria and spectral properties were experimentally explored and computationally simulated by DFT, MP2 and TD‐DFT.

## Results and Discussion

### Synthesis and Characterization

A wide range of methodologies is present in the literature for the synthesis of azomethine‐functionalized compounds based on the dehydration of primary amines with aldehydes or ketones, bearing various steric and electronic properties under mild conditions. The addition of dehydrating agents to reaction mixture was described on several occasions, since water is produced that could inhibit the reaction to some extent.^[1c]^

**Scheme 1:**
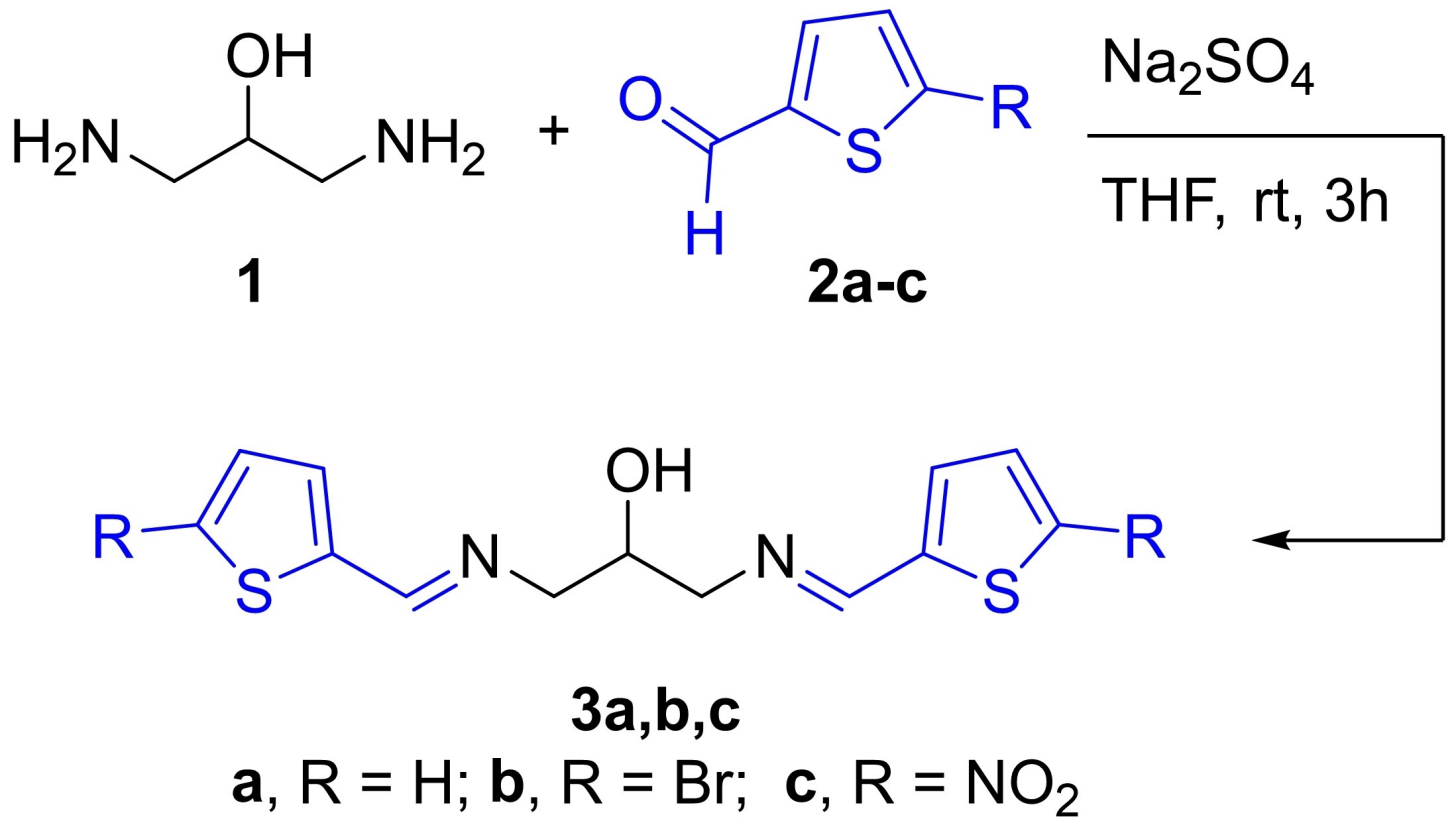
Condensation of 2‐hydroxy‐1,3‐propanediamine (**1**) and 2‐thienylcarboxaldehyde derivatives (**2 a**–**c**)

The new bisazomethine derivatives (**3 a**, **3 b** and **3 c**) were synthesized from the condensation reaction of 2‐hydroxy‐1,3‐propanediamine (**1**) with two molar equivalents of 2‐thienylcarboxaldehydes (thiophene‐2‐carboxaldehyde (**2 a**), 5‐bromothiophene‐2‐carboxaldehyde (**2 b**) and 5‐nitrothiophene‐2‐carboxaldehyde (**2 c**)) in tetrahydrofuran as a solvent at ambient temperature and in the presence of anhydrous sodium sulfate. High‐yield products were isolated and purified by column chromatography to make sure about the purity. Compound **3 a** was identified as oily‐like product, whereas light green and dark brown solids were observed respectively for **3 b** and **3 c**. All products were solely soluble in various polar aprotic and non‐aprotic solvents and to a certain extent in less polar solvents, including acetone, ethanol, DMF, THF, DMSO and dichloromethane. On the other hand, characterization of the new compounds was achieved spectroscopically, illustrating the anticipated products, Scheme 1.

Though the three bisazomethine compounds are sharing a similar backbone and not structurally complicated, they are electronically different. The ^1^H‐NMR measurement in deuterated chloroform displayed all resonances for the three derivative in a very close range. For instance, two sets of a doublet of doublets of doublets (ddd) were observed in the range of 3.63–3.78 and 3.74–3.87 ppm, assigned for the two methylene groups. However, a triplet of triplets signal appeared at the range of 4.14–4.23 ppm was ascribed for the sole aliphatic CH fragment. The three thienylic protons in **3 a** were observed as doublets of doublets (dd) at 7.06, 7.30 and 7.39 ppm. In **3 b**, the two thienylic protons were adjacently observed as doublet (7.02 and 7.02 ppm), while the two signals of **3 c** were detected with wide separation, 7.24 and 7.87 ppm. The close and great distance between these two thienylic protons in **3 b** and **3 c**, respectively, could be rationalized to electronic influence of the Br and NO_2_ groups on the thiophene ring. Moreover, the CH peaks of the azomethine functional moiety were noticed as anticipated as a triplet resonance in the range of 8.27 to 8.42 ppm in the three compounds, Figure S1, S3 and S5.

**Scheme 2:**
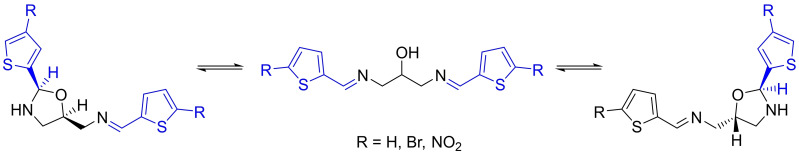
Generation of a mixture of the *cis*‐ and *trans*‐oxazolidines in diastereomeric pairs.

In addition to the peaks of the bisazomethines, the ^1^H‐NMR spectra of the crude products and column‐purified products in CDCl_3_ displayed minor uncharacterizable signals and unrelated to the reactants. The irrelevant resonances were constantly noticeable and possessed varying intensities. These results could point out that the observed additional signals are originated from side‐products resulting from ring‐chain tautomerism to the major products in deuterated chloroform. Either azomethine moiety would undergo intramolecular attack by the hydroxyl group to sp^2^‐hybridized carbon of C=N, leading to the formation of a mixture of the *cis*‐ and *trans*‐oxazolidines in diastereomeric pairs, Scheme 2. The tautomerism of the 2‐hydroxybisazomethineimine‐oxazolidine has been described for a close system by Crumbie and coworker,^[9]^ though the generation of the oxazolidine derivatives is a disfavored 5‐*endo*‐trig route in reference to Baldwin's rules.^[10]^

**Figure 2:**
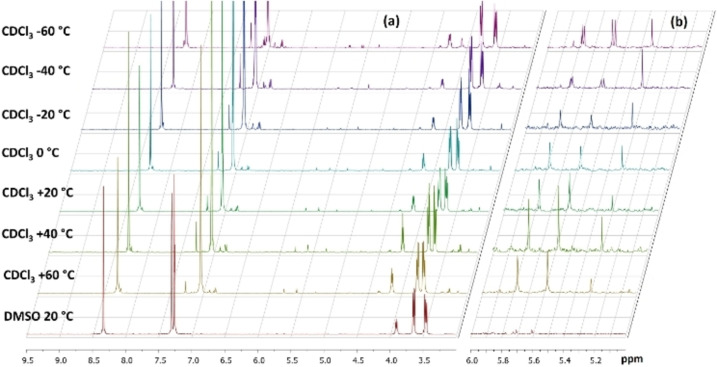
Variable temperature ^1^H‐NMR investigation of **3 b** (a) and expanded section from 5–6 ppm of the spectra.

At this point, variable temperature ^1^H‐NMR investigation was performed to one of the bisazomethine derivatives, namely **3 b**. The existence of these signals was quite stable at different temperature ranging from −60 to +60 °C. As illustrated in Figure 2a, no dramatic changes took place, except the splitting of some peaks were obvious at temperature less than or equal to −40 °C, Figure 2b. Surprisingly, further investigations disclosed that these extra signals were disappeared, once the measurement was accomplished in deuterated DMSO. This led us to suggest that suppression of the tautomeric interconversion takes place through hydrogen bonding between the 2‐hydroxyl group and DMSO molecules. Hence, the competition of intramolecular hydrogen bonding with the azomethine moiety is most likely restrained and forbidding the ring‐chain tautomerism.^[11]^

The ^13^C‐NMR of the three compounds demonstrated all the matching thienylic, aliphatic and iminic C signals in the spectra at the expected area of chemical shifts. For example, the CH fragment displayed a signal around 70 ppm, while both CH_2_ groups were shown in a single peak at 64 ppm. The four theinylic carbons resonance were obvious in the range of 129–152 ppm; the remaining carbon signal of the azomethine group appeared around 156 ppm in all compounds, Figure S2, S4 and S6.

The LC‐MS (ESI) spectra of **3 a** and **3 c** afforded the main mass‐to‐charge ratio (*m*/*z*) peaks at a *m*/*z*=278.9 [M]^+^ and *m*/*z*=370.0 [M+2H]^+^, respectively. Whereas, **3 b** displayed the typical three major signals (*m*/*z*: 438.8 [M]^+^, 436.8 [M]^+^, 434.8 [M]^+^), since bromine possesses two major isotopes (^79^Br and ^81^Br) in almost 1 : 1 part.^[12]^ Remarkably, all compound share some akin fragments, including 288 [M]^+^, 244 [M]^+^, 102 [M]^+^ and 74 [M]^+^, see Figure S7–S9. Furthermore, the expected molecular formulas were characterized employing elemental analysis (CHN), resulting a range of reasonable values, which matched very well with the anticipated structures.

**Figure 3:**
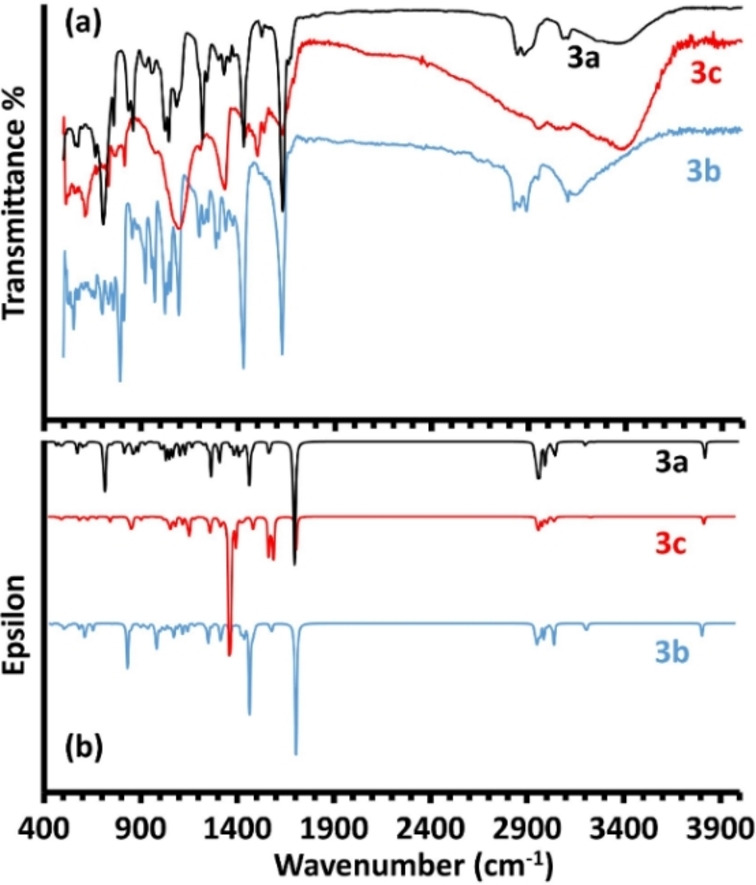
Experimental FTIR (a) and DFT‐IR (b) of bisazomethine compounds.

The experimental IR spectra of the desired products are exemplified in Figure 3a. The condensation reaction led to the evanescence of the N−H stretching bands, while the broad O−H vibration was still obvious at around 3390 cm^−1^, that is expected to be involved in the formation of hydrogen bonding with C=N group in the new compounds. The presented vibrational bands in the area of 3101 to 2917 cm^−1^ were respectively ascribed to the distinctive aliphatic and thienylic C−H stretching frequencies, though they were initially presented in the aldehyde and amino alcohol starting materials. Further indications of the formation of azomethine functional moiety were illustrated clearly by the strong band in the area of 1629 to 1635 cm^−1^ for the absorption of C=N bond; stretching vibrations for other functional modes belong to C−N, C−O, C−C and C=C were displayed in the absorption spectra.^[13]^ On the other hand, the calculated DFT frequencies of the new products (calculated in parallel with optimization at the same level of theory) revealed a worthy level of consistency with experimental consequences, as shown in Figure 3b.

### Thermochemistry and relative stability

**Figure 4:**
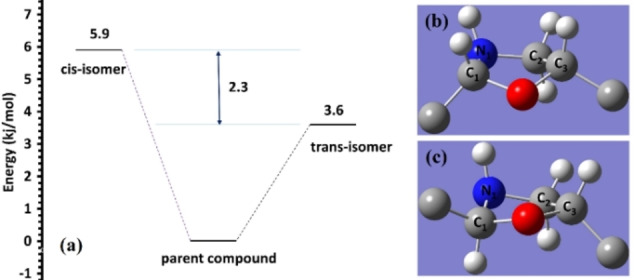
Energy level diagram of the parent compounds (**3 b**) and it tautomers. Energy values represent the enthalpies in kJ/mol at 298 K (a). Ring strain of cis‐isomer (b) and trans‐isomer (c).

To enhance our understanding into the thermodynamics of the *cis‐trans* tautomerism of the parent structure (**3 b**), we calculated the enthalpies (ΔH_298_), Gibbs free energies (ΔG_298_) and the entropies (ΔS_298_) of the tautomerism at 298 K at the MP2/6‐311++G(d,p) level of theory and IEFPCM/CHCl_3_ as solvation model, as explained in the visualizations and computations section. Table 1 displays the thermochemical parameters of the *cis‐* and *trans‐*isomers in relative to the parent molecule in units of kJ/mol and J/mol K. The differences in the reaction enthalpy between the *cis* and *trans* in respect to the parent molecule are equal to 5.9 and 3.6 kJ/mol, respectively. As anticipated, the *trans*‐isomer is 2.3 kJ/mol fewer than the value of *cis*‐isomer due to its less steric interaction between the substituents, Figure 4. In terms of Gibbs free energies, the differences are 11.3 and 11.9 kJ/mol for the *cis* and *trans*, respectively. Since the equilibrium constant is related to ΔG_298_ by the relation ΔG_298_=−RTlnK, the values obtained in this work conclude that the two tautomers are in equilibrium.^[14]^ Moreover, Table 1 demonstrates the entropy values of tautomerism in negative, displaying −18.1 and −28.0 J/mol K, respectively for *cis*‐ and *trans*‐isomers. These figures are in agreement with the structures of molecules; since the two tautomers possess ring strains (Figure 4b,c), they have lower entropies than the parent molecule. To illuminate the difference in entropy of the two tautomers, the dihedral angle (C_1_‐N_1_‐C_2_‐C_3_) for the *trans*‐isomer measured 32.5°, while the *cis*‐isomer displayed 16.6°. This infers that the *trans*‐isomer has a higher ring strain and therefore lower entropy.

**Table 1:** Enthalpies, Gibbs free energies and entropies of the tautomers of **3 b**, obtained at B3LYP/6‐311G++(d,p) and MP2/6‐311++G(d,p) level of theory.

Physical parameter	*cis*‐isomer	*trans*‐isomer
ΔH_298_ (kJ/mol)	5.9	3.6
ΔG_298_ (kJ/mol)	11.3	11.9
ΔS_298_ (J/mol K)	−18.1	−28.0

### Electronic absorptions, electronic transitions and optical bandgap estimation

**Figure 5:**
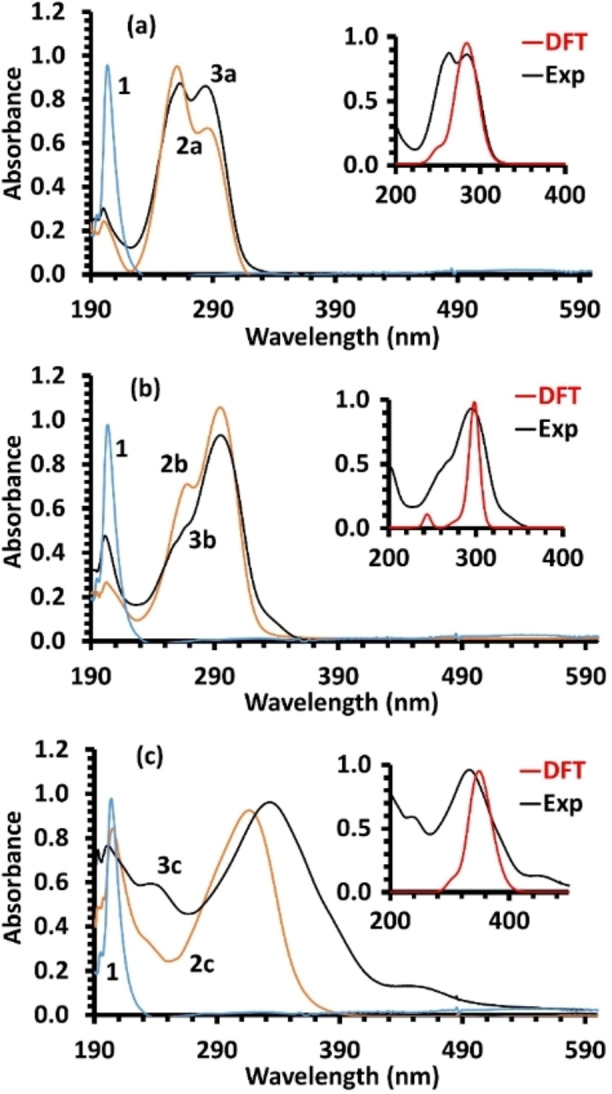
UV/Vis of starting materials and desired compounds, the insets demonstrate the experimental and DFT calculated bands, of **3 a** (a), **3 b** (b) and **3 c** (c).

In the spectral region of 190–800 nm, the UV/Vis measurement of the reactants and azomethine compounds were measured in ethanol. The general outlook of the spectra of the desired derivatives were not comparable, although they possess relatively identical scaffold with only a difference on the 5‐position of thiophene ring. Thus, the substituents on the thienyl ring clearly influenced the electronic arrangement of the boundary orbitals. Initially, the 1,3‐diamino‐1‐propanol exhibited an absorption band in the ultraviolet zone with λ_max_=204 nm, which ascribed to n–σ* transition, whereas the thienylcarboxaldehyde **2 a** showed three bands with λ_max_=201, 261 and 286 nm. These absorptions were reproduced in the product (**3 a**), 200, 263 and 284 nm, but with nearly equivalent intensities in the last two maxima than in **2 a**. Such electronic absorptions point toward a minor hypsochromic effect (blue shift) and could be rationalized to n‐σ*, π–π* and n–π* transitions, Figure 5a. The thienylcarboxaldehyde **2 b** principally revealed two bands at λ_max_=203 and 295 nm and the ultimate displayed a shoulder at 268 nm, however, the azomethine **3 b** displayed as well two broad absorptions with maximum at 202 (n–σ*) and 293 (π‐π* and n–π*) nm. Notably, the shoulder was fused with the major band because of the minor hypsochromic effect, Figure 5b. Moreover, the thienylcarboxaldehyde **2 c** exposed a maximum absorption at 205 nm, overwhelming a shoulder at 235 nm; a main absorption at λ_max_=316 nm. It's functionalized derivative (**3 c**) revealed two strong electronic absorptions with λ_max_=195 nm possessing a shoulder at 236 nm and an intense absorption band at 332 nm along with low‐intensity broad band at 455 nm. The main band shift refers to a bathochromic effect (red shift) and these transitional bands were ascribed similarly to n–σ*, π–π* and n–π*, as shown in Figure 5c. In general, the detected low hypsochromic shift in **3 a** and **3 b** and bathochromic shift in **3 c** confirm the formation of the azomethine‐functionalized compounds via dehydration reaction.

In order to explore the foundation of the electronic transitions of the azomethine derivatives, the vertical excitation energies for the first twelve states were calculated by harnessing the optimized ground state structures in real‐time time‐dependent density functional theory (TD‐DFT). After benchmarking a number of exchange correlation functionals, we found that the HSEH1PBE^[15]^ was the functional of choice to produce satisfactory outcomes in reference to the experiments. Additionally, the 6‐311++G(d,p) was used as basis set and solvation model was achieved in ethanol using the Polarizable Continuum Model (PCM) via the Integral Equation Formalism version (IEFPCM). The mode permits the creation of solute cavity over a sequence of overlapping domains.^[16]^ The insets in Figure 5a–c illustrate a good agreement between the practical UV/Vis and calculated TD‐DFT outcomes. The focal excitation afforded from these calculations allowed a smooth elucidation of the experimental results; Table 2 shows major electronic transitions, including their energies and oscillator strengths.

**Table 2:** Oscillator strengths and energies of the fourth highest electronic transitions in each compound.

Pr.	λ (nm)	Osc. Stren.	ΔE (eV)	Major Contributions
**3 a**	292	0.29	4.244	HOMO‐>LUMO (95 %)
	284	0.16	4.361	H‐4‐>L+1 (30 %), H‐1‐>LUMO (23 %), HOMO‐>L+1 (32 %)
	275	0.49	4.508	H‐1‐>LUMO (19 %), H‐1‐>L+1 (74 %)
	272	0.18	4.554	H‐1‐>LUMO (31 %), H‐1‐>L+1 (15 %), HOMO‐>L+1 (48 %)
**3 b**	309	0.38	4.011	HOMO‐>LUMO (97 %)
	301	0.29	4.117	H‐1‐>LUMO (52 %), HOMO‐>L+1 (43 %)
	293	0.51	5.234	H‐1‐>LUMO (13 %), H‐1‐>L+1 (81 %)
	246	0.06	5.037	H‐5‐>LUMO (83 %)
**3 c**	365	0.32	3.396	HOMO‐>LUMO (94 %)
	344	0.58	3.602	H‐3‐>LUMO (10 %), H‐1‐>LUMO (20 %), H‐1‐>L+1 (54 %)
	340	0.10	3.644	H‐1‐>LUMO (52 %), H‐1‐>L+1 (11 %), HOMO‐>L+1 (24 %)
	307	0.08	4.040	H‐5‐>LUMO (71 %), H‐5‐>L+1 (14 %)

Such transitions are in an adequate harmonization with the experimental absorption bands of the UV/Vis spectra.^[17]^ For illustration, the maximum absorption at 284 nm in **3 a** corresponds to the following major contributions: H‐4→L+1, H‐1→LUMO, HOMO→L+1 and HOMO→LUMO, transitions (284 and 292 nm) with oscillator strength of 0.29 and 0.16. Whereas, the second experimental band at 263 nm would agree to some extent with the two transitions at 272 and 275 nm, which matches to H‐1→LUMO, H‐1→L+1 and HOMO→L+1 transitions with strength of 0.18 and 0.49, in respect to wavelengths. The wide experimental band at 295 nm in **3 b** corresponds principally with the three transitions at 293, 301, and 309 nm (with strength of 0.51, 0.29 and 0.38, respectively), resulting from H‐1→LUMO, H‐1→L+1, HOMO→L+1 and HOMO→LUMO, transitions. The obvious minor transition at lower wavelength with oscillator strength of 0.06 is ascribed to H‐5→LUMO transition with high percentage. Furthermore, the broad absorption with λ_max_=333 nm in compound **3 c** is expected to result from four major transitions at 307, 340, 344 and 365 nm with oscillator strength equal to 0.08, 0.10, 0.58 and 0.32, respectively. Again, these are subsequent of H‐5‐>LUMO, H‐5‐>L+1, H‐1→LUMO, H‐1‐>L+1, HOMO→L+1, H‐3→LUMO HOMO→LUMO transitions in different percent values as indicated in Table 2.

**Figure 6:**
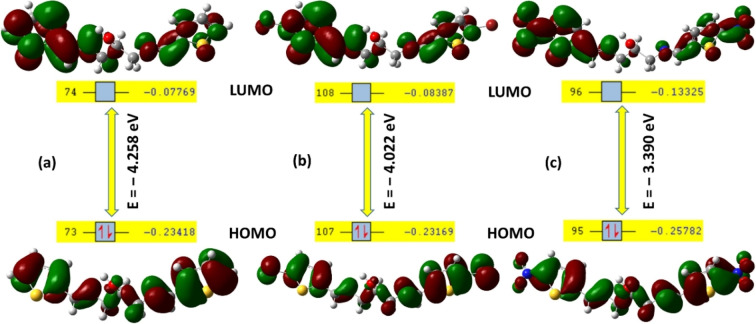
Frontier molecular orbitals and energy values of **3 a**–**c**.

It is worth mentioning that the contour plots of frontier molecular orbitals, explicitly HOMO and LUMO, are important to investigate the localization and delocalization of the electron density on all three structures. High level of similarity is notable among the structure and particularly between compounds **3 a** and **3 b**, since their molecular orbitals possess a close range of energy values, (HOMO: −0.23418 and −0.23169; LUMO: −0.07769 and −0.08387 in au unit) Figure 6a,b. The electron density in HOMO is almost localized over the C=C and C=N bonds of the molecular structures. On the other hand, the orbital lobes in LUMO are delocalized on the unsaturated moieties, including the thienyl rings and the azomethine functional groups. It is notable that the atomic orbitals in **3 b** are localized on the Br substituents in both HOMO and LUMO. Conversely, Compound **3 c** displayed that the electron density of NO_2_ group is attracted towards the C=C bond of thiophene rings in HOMO; delocalized with the electron density of the conjugated system in LUMO, Figure 6c. Although, these three compounds are symmetric, the electron delocalization is mostly distributed on one side of the structures. This could be rationalized to tendency for the formation of an H‐bond between the OH and C=N groups.

The electron distribution of frontier molecular orbitals could deliver valuable estimation on the solvatochromic effect for compound **3 b** and **3 c**, though the HOMO and LUMO of **3 a** and **3 b** are almost identical. It is obvious that the Br substituents are excluded from the conjugated electron distribution in LUMO, whereas the nitro groups in **3 c** are included in the delocalization of the orbital lobes in LUMO, i. e. extending the electron distribution. The high delocalization during the HOMO‐LUMO electron transition is expected to play a role in charge‐transfer character of **3 c** in different environment, namely, the presence of solvent molecules.^[18]^ This estimation was experimentally pursued and discussed in the next section.

Since HOMO signifies the orbital that turns as an electron donor and LUMO is the orbital that represents as the electron acceptor, the difference between the energy levels delivers beneficial information on the structure stability and the global chemical reactivity. Hence, the ΔE were figured out to be −4.258, −4.022 and 3.390 eV, respectively for **3 a**, **3 b** and **3 c** (Figure 6). Such energies (*E_g_*) are essential value in order to promote an electron from the valence band (HOMO) to the conduction band (LUMO).

To experimentally measure the optical bandgap for estimating the photochemical and photophysical properties of the new compounds for the purpose of optoelectronic devices, a quantification of absorption‐based spectra is frequently utilized approach for this purpose. A practical technique for assessing the bandgap energy was defined by Tauc and coworker employing the absorption spectra^[19]^ through the presumption that the coefficient of energy‐dependent absorption (*α*) can be specified by equation 1.

(1) $$\left(\alpha \;\cdot \;h\nu \right){}^{1/\gamma }=B\left(h\nu -E{}_{g}\right)$$

In which, *h* is the Planck constant, B is a constant, and *ν* is a frequency of a photon. Moreover, γ relies on the transition of an electron; equal to 1/2 or 2, respectively for substance with direct or indirect permitted transitions.^[20]^ On the other hand, the assessment of α could be performed using the Beer‐Lambert's relation, equation 2.

(2) α=2.303A/d

**Figure 7:**
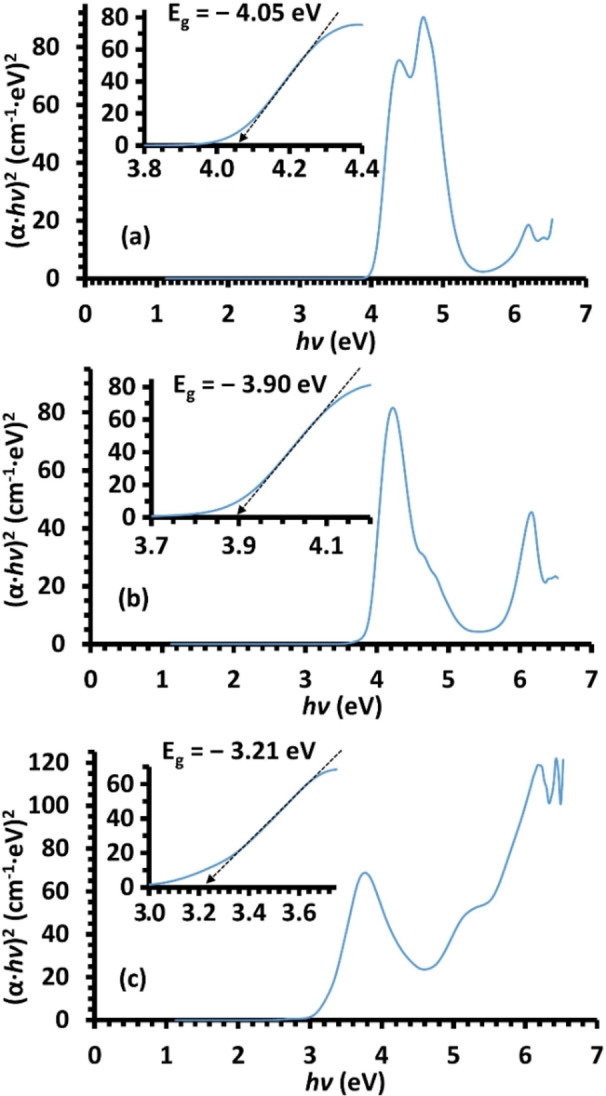
Tauc charts extracted from UV/Vis spectra of **3 a**–**c**, the insets illustrate the evaluated optical bandgap.

In which, A is the absorbance of the measured sample and d is the distance of the cell in cm.^[21]^ Therefore, the experimentally recorded UV/Vis absorptions (A and λ) of all compounds (**3 a**–**c**) were separately transformed to reflectance‐absorption curves following equations 1 and 2. Once the (*α*⋅*hν*)^2^ is plotted versus energy of photon, the *E_g_* is considered by a region showing a steep and linear increase of absorbed light with increasing energy. By extrapolating this linear area to the X‐axes, assessment of the energy of optical bandgap for each compound is obtained. Hence, the optical gaps were found to be: *E_g_* =−4.05 eV, *E_g_* =−3.90 eV and *E_g_* =−3.21 eV, respectively for **3 a**, **3 b** and **3 c**, as illustrated in Figure 7. Notably, these numbers are fairly around the outcomes of TD‐DFT calculations, Figure 6. Furthermore, these energy values are in the domain of wide‐bandgap materials (2–4 eV); possessing electronic properties placed between insulators and conventional semiconductors.^[22]^ Based on these results, the three azomethine‐functionalized derivatives could be employed as substrates for organic nonlinear optical materials to yield a nonlinear response, as they displayed valuable interactivity with light.[^21b^, ^23^]

### Surface Analysis and Solvatochromism

**Figure 8:**
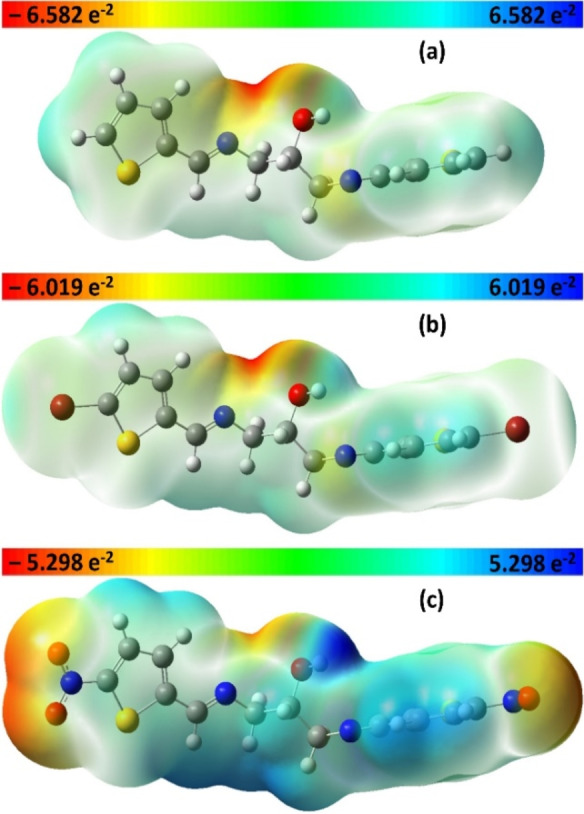
MEP visualization in transparent format of **3 a** (a), **3 b** (b) and **3 c** (c).

The optimized ground state geometries in vacuum revealed that the three thienyl rings are almost perpendicular to each other, which could play a significant role in structure stability in the crystal lattice. The electronic distributions are relatively similar on the three structures, although, the variance is situated on the 5‐position of thienyl moieties. In order to link the molecular stability with molecular electronic structure, Figure 8 displays the molecular electrostatic potential, where the nucleophilic and electrophilic centers are shown by the potential decrease. The electron density is mainly fluctuating from red, yellow, green, white to blue. In each compound, the alcoholic functionality (O atom) is highlighted in red as it is considered as nucleophilic group; however, the two azomethine moieties clearly represent a nucleophilic center on the N atom and an electrophilic center on the C atom. Furthermore, the O atoms of NO_2_ moieties in **3 c** retain the greatest nucleophilic sites; the electrophilic sites are highly located on the C and S atoms. The rest of scaffold atoms (H) is demonstrated by blue for electrophilic centers. These variations are anticipated to stabilize the molecular frame through the non‐ classical C−H⋅⋅⋅π stacking and classical hydrogen bonding, as well to interact with diverse external molecules (solvent), leading to a probable change in the absorption spectra.^[24]^

Therefore, the presence of a compound in a solvent medium will affect its electronic system and causing a perturbation, resulting from the noncovalent interactions between the solvent and compound molecules.^[25]^ The molecular orbitals of the engaged molecules will be included in the electronic absorption, which is driven to alter its maximum. In addition, color change of the solution is considered as a subsequent step and will take place when a substantial difference in the solvation energy is accomplished.

In order to investigate the consequence of solvents on maximum absorption and to estimate the ground and excited states, solvatochromism is usually considered for these purposes.^[26]^ The ground state is more stable in less polar solvents than the excited state and will lead to negative solvatochromism (hypsochromic shift). Whereas, the excited state is relatively more robust in more polar solvents and will conduct positive solvatochromism (bathochromic shift).^[27]^

**Figure 9:**
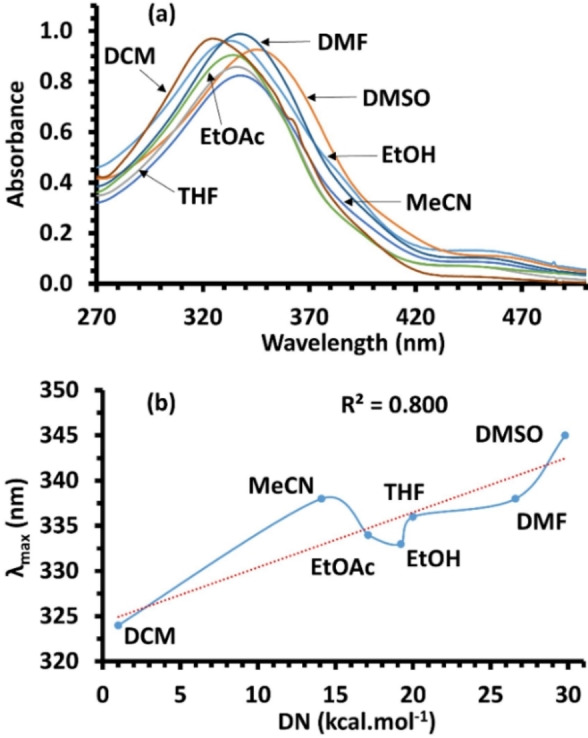
Solvatochromic influence of **3 c** in various solvents (a) and relation between maximum absorption and Gutmann's solvent DNs (b).

To appraise the solvatochromic effect of the synthesized azomethine compounds, the power of various solvents on the absorption spectra was followed under ambient conditions. The experiments were achieved utilizing solvents with different polarity, including dimethylformamide, acetonitrile, ethyl acetate, ethanol, dimethyl sulfoxide, dichloromethane and tetrahydrofuran. The **3 a** and **3 b** were ruled out from the detailed study, since no valuable change in the maximum absorption was noticed using different solvents. Whereas, a significant shifting in the solvatochromism was determined in the area of 270–500 nm for compound **3 c**, as shown in Figure 9a. The λ_max_ in ethanol (333 nm) was shifted toward blue area when dichloromethane is used, λ_max_=324 nm. While, a progressive shift to the red region was detected by increasing the polarity of the employed non‐protic solvent, principally with DMSO and DMF. Thus, greater absorption was noticed with DMSO (λ_max_=345 nm), referring to a 21 nm of red shift in respect to DCM. This performance could be ascribed to the aforementioned facts that the π–π* excitation state is very stable in polar aprotic solvents; in turn leads to depress the bandgap energy. Furthermore, the experimentally measured maximum absorption and Gutmann's donicity numbers (DN)^[28]^ were set in solvation relation, showed a sense of progressive linear manner with correlation coefficient (R^2^) of 0.800. This fluctuation was slightly resulting from ethanol and acetonitrile solvents that could be resonated to a weak interaction between the solvent and solute molecules, Figure 9b.

On the other hand, we examined the solvent impact on the first three excited states of compound **3 c** using TD‐DFT method and the HSEH1PBE functional. As seen in Table 3, the computed excitation energies displayed minimum values of 3.391, 3.590 and 3.630 eV in DMSO and maximum values of 3.440, 3.694 and 3.728 eV in CCl_4_ for *S*_1_, *S*_1_ and *S*_3_ respectively. The estimated energy difference of 49 (*S*_1_), 104 (*S*_2_) and 98 (*S*_3_) meV and shifting in transitions of about 6 (*S*_1_), 9 (*S*_2_) and 9 (*S*_3_) nm indicated the influence of solvent molecules on the electronic transitions.

**Table 3:** Solvent influence on the first three low‐lying excited states.

States eV (nm)	Solvents					
	DMSO	MeCN	EtOH	DCM	THF	CCl_4_
*S*_1_	3.391 (366)	3.395 (365)	3.396 (365)	3.405 (364)	3.409 (364)	3.440 (360)
*S*_2_	3.590 (345)	3.599 (344)	3.602 (344)	3.616 (343)	3.624 (342)	3.694 (336)
*S*_3_	3.630 (342)	3.644 (340)	3.644 (340)	3.653 (339)	3.661 (339)	3.728 (333)

## Conclusion

Three new compounds of 1,3‐bis(((*E*)‐thiophen‐2‐ylmethylene)amino)propan‐2‐ol were prepared in high yields from the dehydration reaction of 2‐hydroxy‐1,3‐propanediamine and 2‐thienylcarboxaldehydes. The expected products were spectroscopically identified and further explored by DFT calculations.

The ^1^H‐NMR clearly displayed the presence of the parent compounds in tautomerism, which was influenced by the solvent capability to form H‐bond. The calculated ΔH_298_, ΔG_298_ and ΔS_298_ of **3 b** at DFT and MP2 levels of theory supported the equilibrium of the parent compound with two tautomers. The UV/Vis measurement of **3 a** and **3 b** disclosed a slight hypsochromism, while bathochromism is observed with **3 c**, highlighting the formation of azomethine‐functionalized compounds. The origin of the electronic transitions was examined using TD‐DFT. The MEP revealed that the three structural frames possess almost affiliated electronic distributions. Various nucleophilic and electrophilic centers found on the surfaces were anticipated to give high stability through the C−H⋅⋅⋅π interactions and hydrogen bonding, as well to engage with external molecules of solvent. Compound **3 c** exposed a gradual shift of the maximum absorption to the red area when the polarity of solvent was increased; 21 nm of red shifting was observed. In contrast, no significant solvent‐effect was observed in case of **3 a** and **3 b**. The solvation relation was set between Gutmann's donicity numbers and the experimental λ_max_, exhibiting almost positive linear performance with a minor oscillation due to a possible weak interface between the molecules of solute and designated solvents. Finally, the energygap of all products were assessed experimentally using optical absorption spectra following Tauc approach and computationally from TD‐DFT simulation, giving very close figures. These outcomes proposes that the novel structures could be utilized as organic substrates for nonlinear activity, in addition to be utilized as a proper tridentate ligand through the two N and one O atoms.

## Experimental Section

### Materials and Measurements

Reagent quality chemicals and solvents were directly put in use without purification. 2‐hydroxy‐1,3‐propanediamine and anhydrous sodium sulfate were acquired from Alfa Aesar. Thiophene‐2‐carboxaldehyde, 5‐nitrothiophene‐2‐carboxaldehyde, 5‐bromothiophene‐2‐carboxaldehyde, were purchased from Sigma Aldrich. JOEL 600 MHz spectrometer was employed to measure spectra of ^1^H‐ and ^13^C‐NMR with internal standard to the remaining solvent signal, chloroform‐d: δ=7.26 ppm for ^1^H‐NMR; δ=77.16 ppm and δ=39.52 ppm for chloroform‐d and dimethyl sulfoxide‐d_6_, respectively, for ^13^C‐NMR. The described results are only for products with unambiguous and very clear signals in the NMR spectra. All FTIR spectra were measured in liquid and solid states in the zone of 400–4000 cm^−1^ on Bruker ALPHA FTIR spectrometer. Thermo Scientific™ FLASH™ 2000 Organic Elemental Analyzer was put in use to perform the CHN analysis. UV/Vis absorptions were recorded using solvents of UV grade on single beam Agilent 8453 spectrophotometer. Solvents were acetone, acetonitrile, tetrahydrofuran, dichloromethane, ethanol, N,N‐dimethylformamide and dimethyl sulfoxide. The measurement of mass spectra (LC‐MS) was performed employing Triple Quadrupole LC‐MS, Agilent 6460 Spectrometer.

### Computation and Visualization

The computational studies were carried out using DFT/B3LYP functional method, unless otherwise stated,^[29]^ with 311G++(d,p) basis set. Structural optimization was performed employing the Berny algorithm of Gaussian suit of programs, W09 Revision E.01.^[30]^ The simulation of frequencies for all new structures was executed at the same level of theory of optimization. GaussView software was utilized to create the HOMO and LUMO via the MO editor, using the checkpoint file of TD‐DFT calculation. After loading the Checkpoint File, a window popped up and visualization phase can be executed to separately visualize HOMO and LUMO cubes. The HOMO and LUMO energy values were displayed subsequent to each orbital in au unit. Mapping the molecular electrostatic potential (MEP) was accomplished using the checkpoint file via surface and contours from the Results menu to form a cube file for the total electron density. A mapped surface was then created from the cube file for the ESP. Consequently, generation of the surface appeared, further tuning of the MEP map can be performed through the Display Format option in the new window of the View menu. GaussSum software was harnessed to extract the DFT simulated spectra from the TD‐DFT output file.^[31]^

To predict the thermodynamic data and relative stabilities of **3 b** and the tautomers, the structural optimization and vibrational frequencies of the competitors were simulated utilizing the B3LYP functional, 6‐311++G(d,p) basis set and IEFPCM/CHCl_3_ as solvation model. Since MP2 is known to furnish favorable results, it was used with 6‐311++G(d,p) basis set and IEFPCM/CHCl_3_ as a model to estimate the single‐point‐energy. Moreover, in order to calculate the free energy changes of tautomers at 298 K, enthalpies (ΔH_298_) were obtained by adding zero‐point energy correction and thermal correction to enthalpy to the relative energies resulting from MP2.[^14^, ^32^] Similarly, the Gibbs free energies (ΔG_298_) were obtained using the thermal correction to free energy. The Entropies (ΔS_298_) were calculated from the last two parameters from ΔG_298_=ΔH_298_−TΔS_298_.^[14]^

### Synthesis of 1,3‐bis(((*E*)‐thiophen‐2‐ylmethylene)amino)propan‐2‐ol (3 a)

The desired derivative was accomplished following traditional condensation process. In a 50‐milliliter round‐bottom flask, a mixture of 465 mg (5.0 mmol) of 2‐hydroxy‐1,3‐propanediamine dissolved in 10 mL of tetrahydrofuran and 1144 mg (10.0 mmol, 954 μL) of thiophene‐2‐carboxaldehyde in 10 mL of the same solvent was stirred at ambient temperature for 240 min in the presence of one gram of anhydrous Na_2_SO_4_. The solvent was removed under vacuum and the crude outcome was purified by chromatography employing a mixture of hexane/ethyl acetate as eluent, affording green oily‐like compound in 85 % (1.18 g). ^1^H‐NMR (600 MHz, CDCl_3_): δ=8.42 (t, *J*=1.4 Hz, 2H, *H*C=N), 7.39 (dd, *J*=5.0, 1.6 Hz, 2H, C*H*‐thienyl), 7.30 (dd, *J*=3.6, 1.3 Hz, 2H, C*H*‐thienyl), 7.06 (dd, *J*=5.1, 3.6 Hz, 2H, C*H*‐thienyl), 4.20 (tt, *J*=6.9, 4.7 Hz, 1H, C*H*), 3.80 (ddd, *J*=12.3, 4.8, 1.5 Hz, 2H, C*H_2_*), 3.67 (ddd, *J*=12.3, 6.7, 1.3 Hz, 2H, C*H_2_*). ppm. ^13^C‐NMR (151 MHz„ CDCl_3_): δ=156.4 (2 C, H*C*=N), 142.4 (2 C, *C*‐thienyl), 130.9 (2 C, *C*H‐thienyl), 129.2 (2 C, *C*H‐thienyl), 127.5 (2 C, *C*H‐thienyl), 70.9 (1 C, *C*H), 64.6 (2 C, *C*H_2_) ppm. LC‐MS (ESI), *m*/*z*: 278.9 [M]^+^, 244.2 [M]^+^, 185.0 [M]^+^, 140.0 [M]^+^, 102.0 [M]^+^ and 74.1 [M]^+^. Designated IR absorptions (cm^−1^): 3395 *v*_(O−H)_, 3101 *v*_(C‐Hthienylic)_, 2917 *v*_(C‐Haliphatic)_, 1635 *v*_(C=N)_, 1620 *v*_(C=C)_, 1110 *v*_(C−O)_ cm^−1^. Anal. Calcd. for C_13_H_14_N_2_OS_2_: C, 56.09; H, 5.07; N, 10.06. Found: C, 55.90; H, 5.19; N, 10.12. UV/Vis absorption in EtOH, λ_max_=200, 263 and 284 nm.

### Synthesis of 1,3‐bis(((*E*)‐(5‐bromothiophen‐2‐yl)methylene)amino)propan‐2‐ol (3 b)

The aforementioned procedure for the synthesis of **3 a** was put in use, thus, 465 mg (5.0 mmol) of 2‐hydroxy‐1,3‐propanediamine in 10 mL of tetrahydrofuran and 2011 mg (10.0 mmol) of 5‐bromothiophene‐2‐carboxaldehyde in 10 mL of the same solvent was stirred under the same conditions. The crude outcome was purified by chromatography employing a mixture of hexane/ethyl acetate as eluent, affording light green solid compound in 83 % (1.80 g), mp 133–135 °C. ^1^H‐NMR (600 MHz, CDCl_3_): δ=8.27 (t, *J*=1.4 Hz, 2H, *H*C=N), 7.03 (d, *J*=4.0 Hz, 2H, C*H*‐thienyl), 7.02 (d, *J*=3.9 Hz, 2H, C*H*‐thienyl), 4.14 (tt, *J*=6.6, 4.7 Hz, 1H, C*H*), 3.74 (ddd, *J*=12.4, 4.7, 1.5 Hz, 4H, C*H*_2_), 3.63 (ddd, *J*=12.4, 6.6, 1.2 Hz, 4H, C*H*_2_) ppm. ^13^C‐NMR (151 MHz, CDCl_3_): δ=155.5 (2 C, H*C*=N), 144.0 (2 C, *C*‐thienyl), 130.9 (2 C, *C*H‐thienyl), 130.5 (2 C, *C*H‐thienyl), 117.4 (2 C, *C*Br), 70.7 (1 C, *C*H), 64.3 (2 C, *C*H_2_) ppm. LC‐MS (ESI), *m*/*z*: 438.8 [M]^+^, 436.8 [M]^+^, 434.8 [M]^+^, 262.9 [M]^+^, 208.1 [M]^+^, 167.1 [M]^+^, 102.1 [M]^+^ and 74.1 [M]^+^. Designated IR absorptions (cm^−1^): 3390 *v*_(O−H)_, 3098 *v*_(C‐Hthienylic)_, 2912 *v*_(C‐Haliphatic)_, 1633 *v*_(C=N)_, 1623 *v*_(C=C)_, 1115 *v*_(C−O)_ cm^−1^. Anal. Calcd. for C_13_H_12_Br_2_N_2_OS_2_: C, 35,80; H, 2.77; N, 6.42. Found: C, 35.89; H, 2.91; N, 6.50. UV/Vis absorptions in EtOH, λ_max_=202 and 295 nm.

### Synthesis of 1,3‐bis(((*E*)‐(5‐nitrothiophen‐2‐yl)methylene)amino)propan‐2‐ol (3 c)

The aforementioned procedure for the synthesis of **3 a** was put in use, thus, 465 mg (5.0 mmol) of 2‐hydroxy‐1,3‐propanediamine in 10 mL of tetrahydrofuran and 1604 mg (10.0 mmol) of 5‐nitrothiophene‐2‐carboxaldehyde in 10 mL of the same solvent was stirred under the same conditions. The crude outcome was purified by chromatography employing a mixture of hexane/ethyl acetate as eluent, affording dark brown compound in 87 % (1.60 g), mp 145–147 °C. ^1^H‐NMR (600 MHz, CDCl_3_): δ=8.41 (t, *J*=1.4 Hz, 2H, *H*C=N), 7.87 (d, *J*=4.1 Hz, 2H, C*H*‐thienyl), 7.24 (d, *J*=4.5 Hz, 2H, C*H*‐thienyl), 4.23 (tt, *J*=6.5, 4.9 Hz, 1H, C*H*), 3.87 (ddd, *J*=6.1, 4.4, 1.2 Hz, 2H, C*H_2_*), 3.78 (ddd, *J*=12.3, 6.6, 1.4 Hz, 2H, C*H_2_*). ppm. ^13^C‐NMR (151 MHz, CDCl_3_): δ=156.0 (2 C, H*C*=N), 151.8 (2 C, *C*NO_2_), 148.8 (2 C, *C*‐thienyl), 130.3 (2 C, *C*H‐thienyl), 130.2 (2 C, *C*H‐thienyl), 69.4 (1 C, *C*H), 64.6 (2 C, *C*H_2_) ppm. LC‐MS (ESI), *m*/*z*: 370.0 [M+2H]^+^, 288.2 [M]^+^, 244.2 [M]^+^, 102.1 [M]^+^ and 74.1 [M]^+^. Designated FTIR absorptions (cm^−1^): 3395 *v*_(O−H)_, 3100 *v*_(C‐Hthienylic)_, 2939 *v*_(C‐Haliphatic)_, 1629 *v*_(C=N)_, 1625 *v*_(C=C)_, 1360 *v*_(N=O)_, 1112 *v*_(C−O)_ cm^−1^. Anal. Calcd. for C_13_H_12_N_4_O_5_S_2_: C, 42.39; H, 3.28; N, 15.21. Found: C, 42.30; H, 3.44; N, 15.16. UV/Vis absorptions in EtOH, λ_max_=193, 236 and 333 nm.

## Conflict of interest

The author declares no conflict of interest.

1

## Supporting information

As a service to our authors and readers, this journal provides supporting information supplied by the authors. Such materials are peer reviewed and may be re‐organized for online delivery, but are not copy‐edited or typeset. Technical support issues arising from supporting information (other than missing files) should be addressed to the authors.

Supporting Information

## Data Availability

Research data are not shared.
