# Supplementary material for: Solvent Influence on Absorption Spectra and Tautomeric Equilibria of Symmetric Azomethine‐Functionalized Derivatives: Structural Elucidation and Computational Studies
Source: ChemistryOpen. 2022 Feb 22;11(2):e202100237. doi: 10.1002/open.202100237 (PMC8862155; doi:10.1002/open.202100237)
Supplement: Supplementary file 1 — Supporting Information [file OPEN-11-e202100237-s001.pdf]

ChemistryOpen

Supporting Information

Solvent Influence on Absorption Spectra and Tautomeric Equilibria of Symmetric Azomethine-Functionalized Derivatives: Structural Elucidation and Computational Studies

Kifah S. M. Salih*

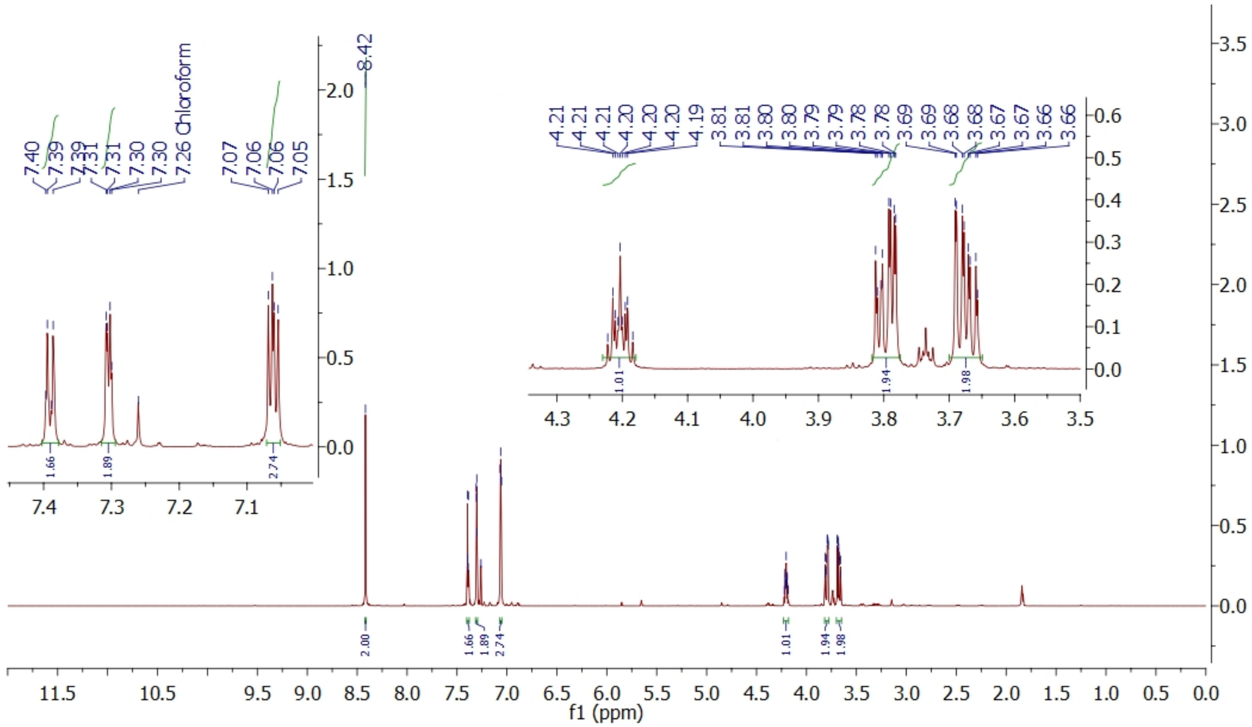

Fig. S1: ^{1}H-NMR spectrum of **3a** measured in $CDCl_3$.

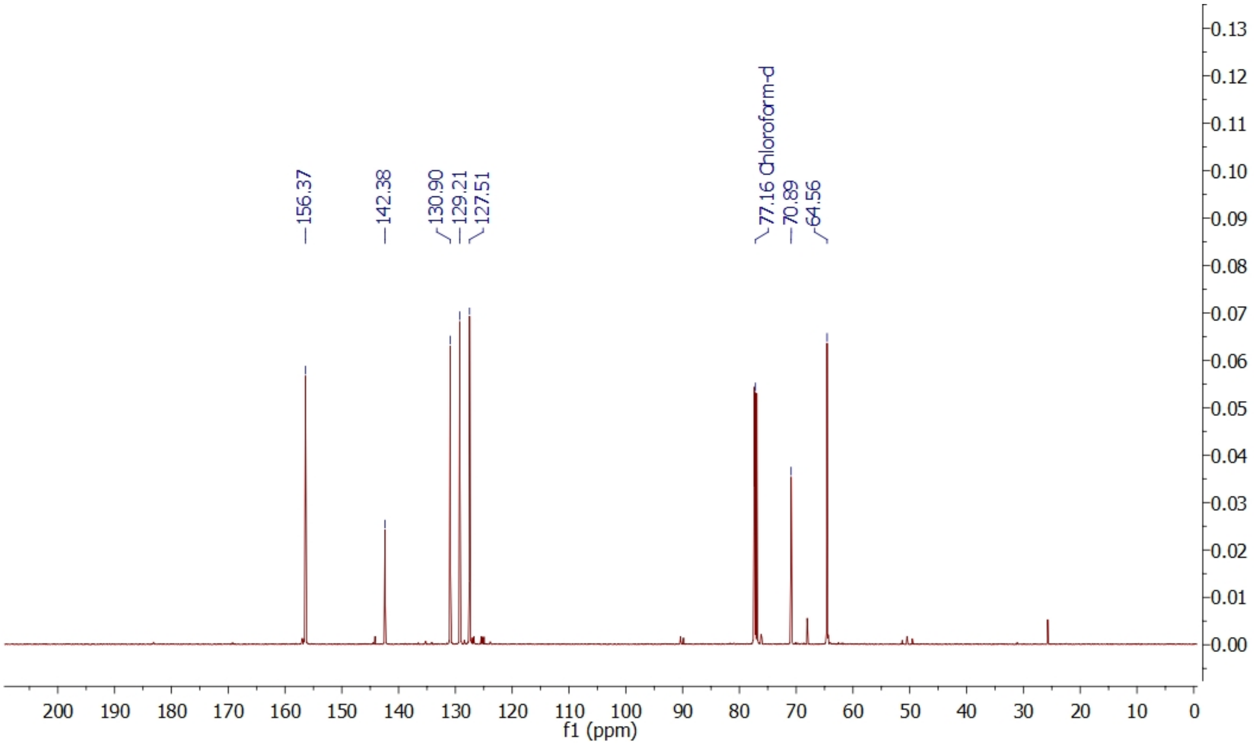

Fig. S2: ^{13}C-NMR spectrum of **3a** measured in $CDCl_3$.

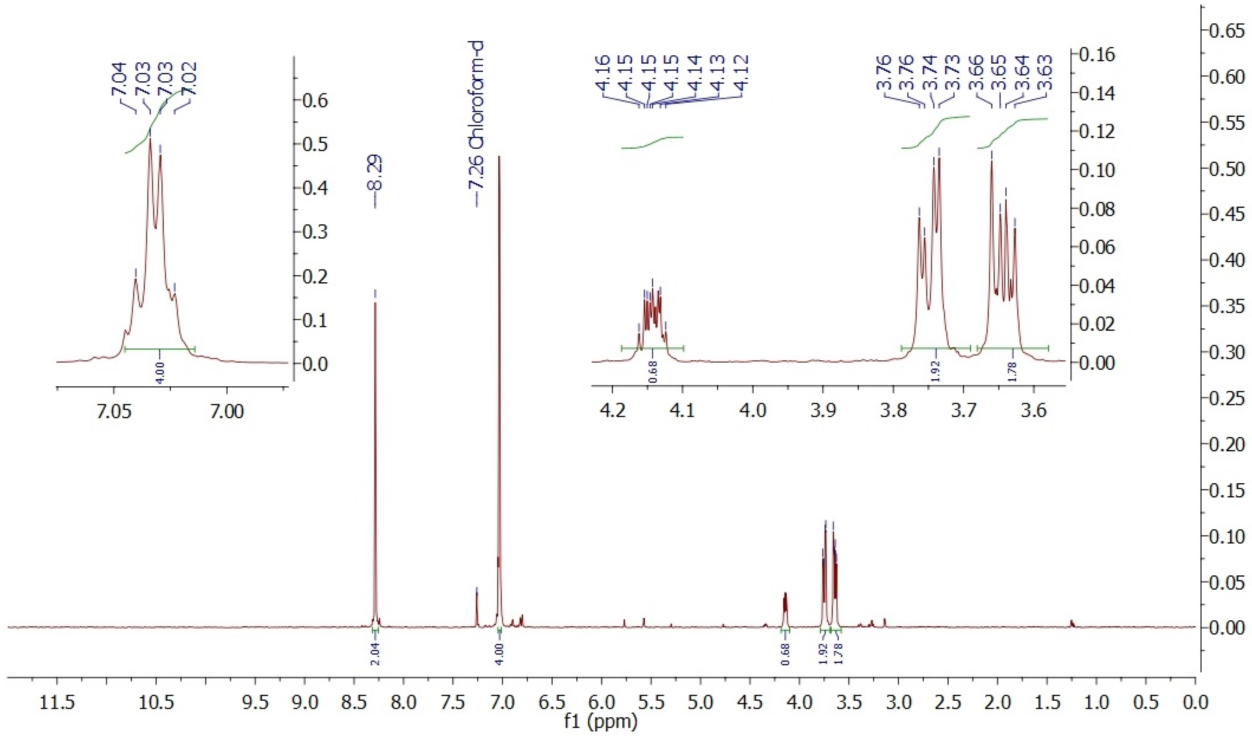

Fig. S3: ^{1}H-NMR spectrum of **3b** measured in $CDCl_3$.

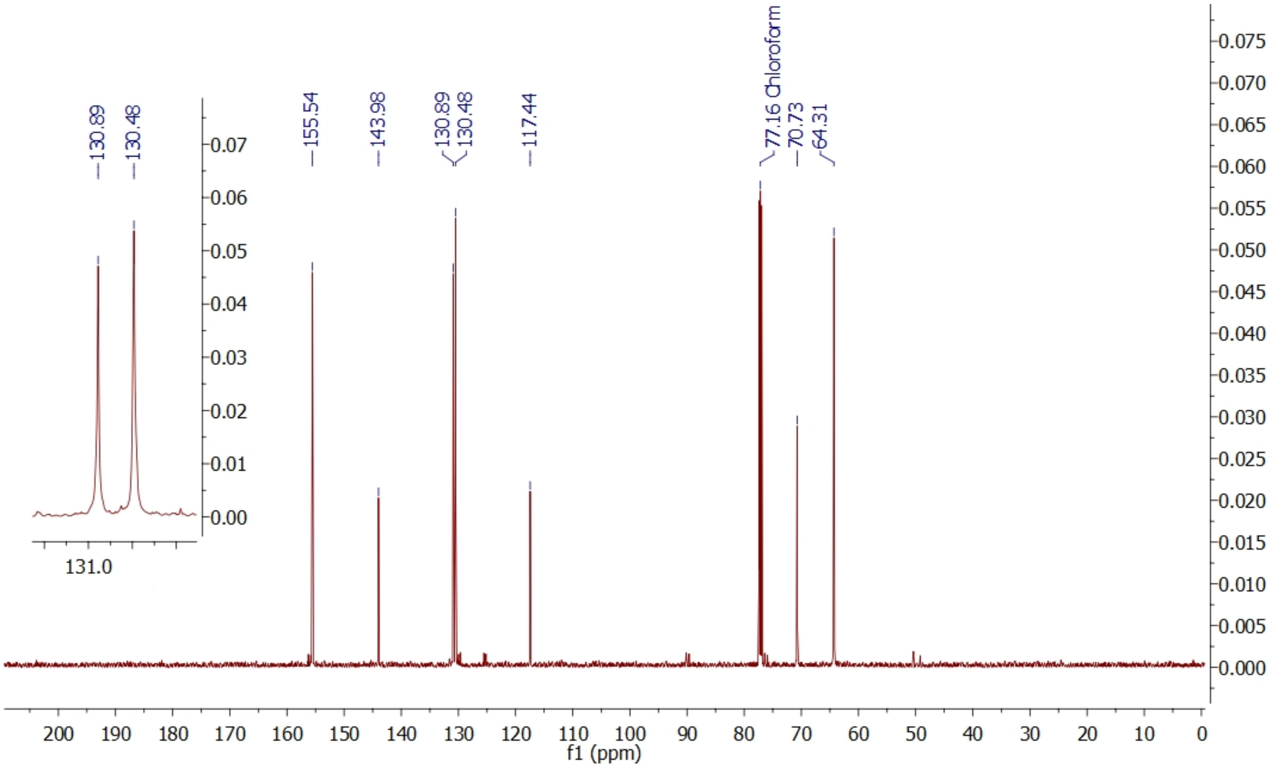

Fig. S4: ^{13}C-NMR spectrum of **3b** measured in $CDCl_3$.

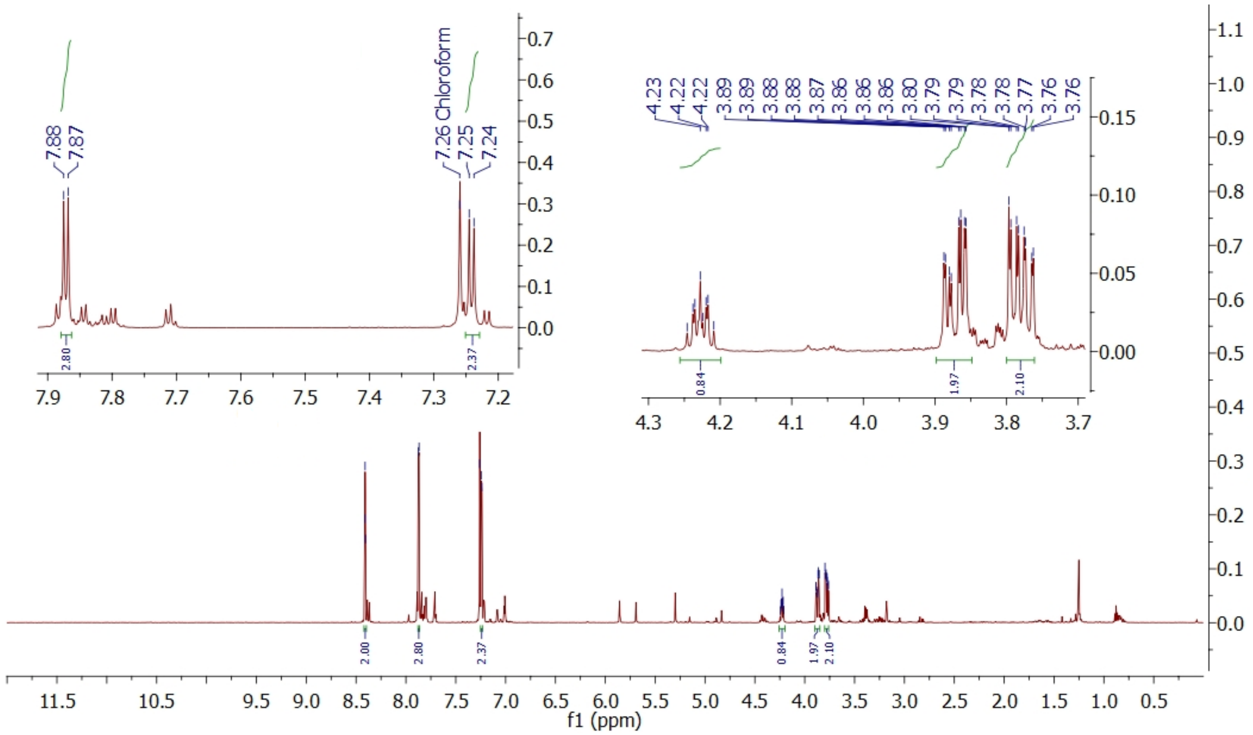

Fig. S5: ^{1}H-NMR spectrum of **3c** measured in $CDCl_3$.

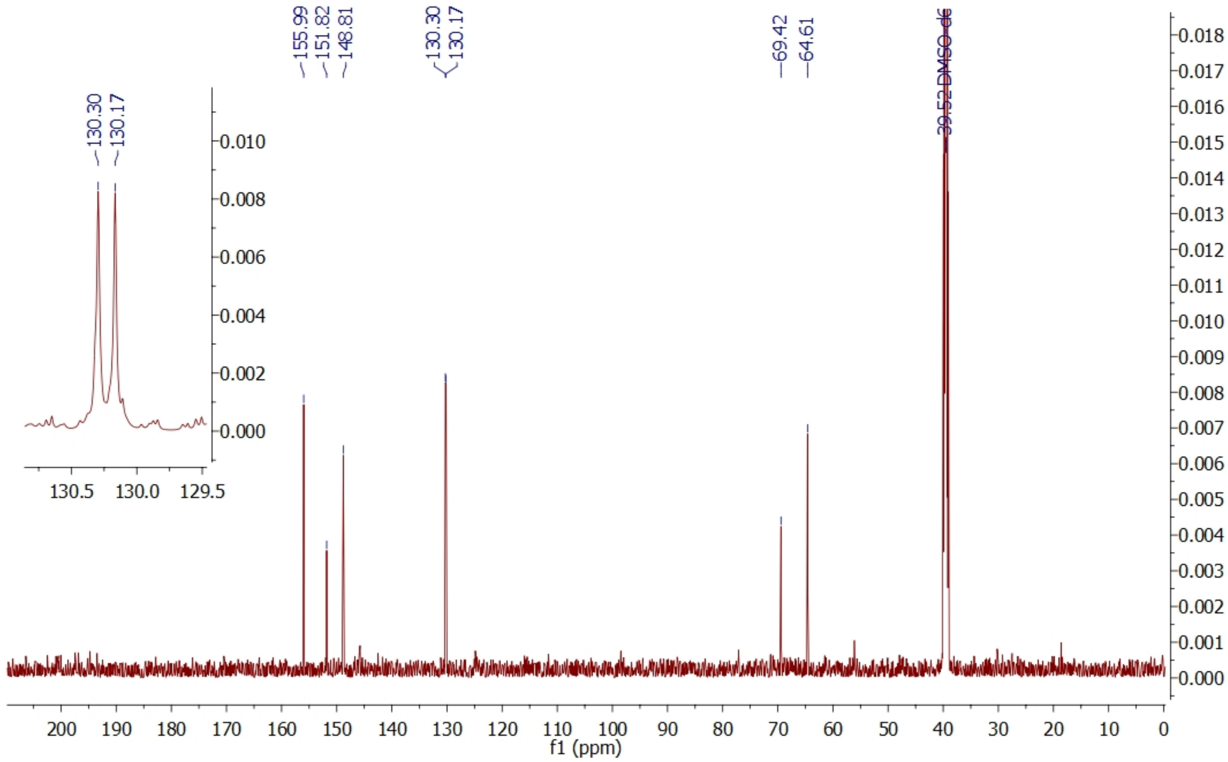

Fig. S6: ^{13}C-NMR spectrum of **3c** measured in DMSO-d_6.

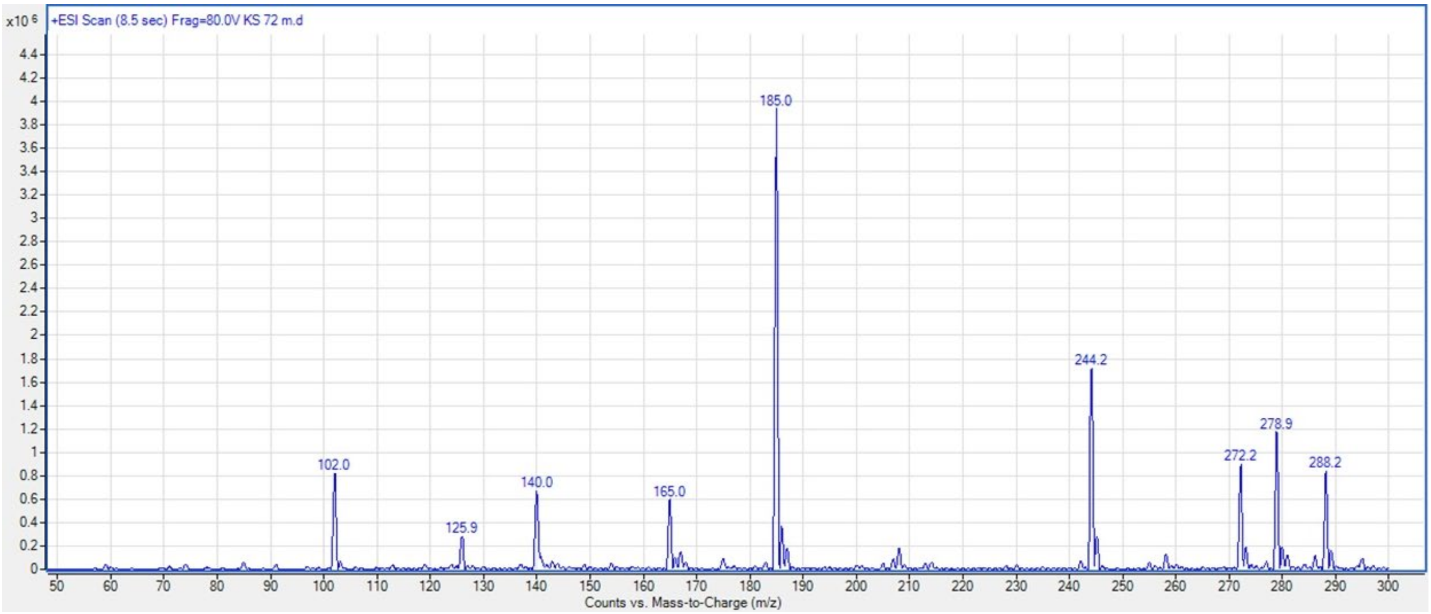

Fig. S7: LC-MS spectrum of **3a**.

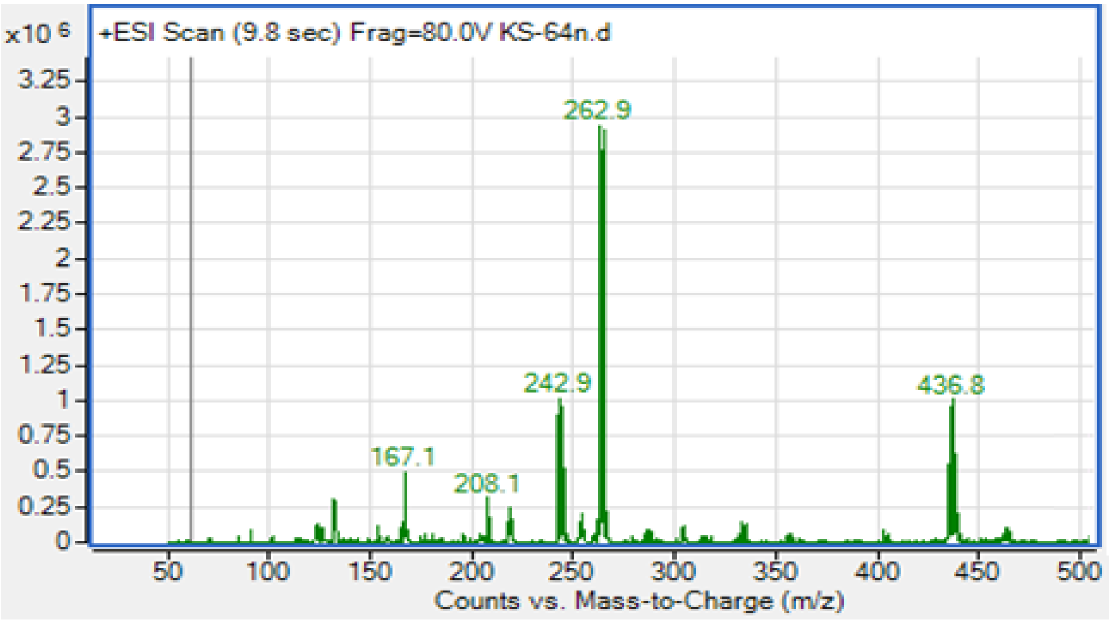

Fig. S8: LC-MS spectrum of **3b**.

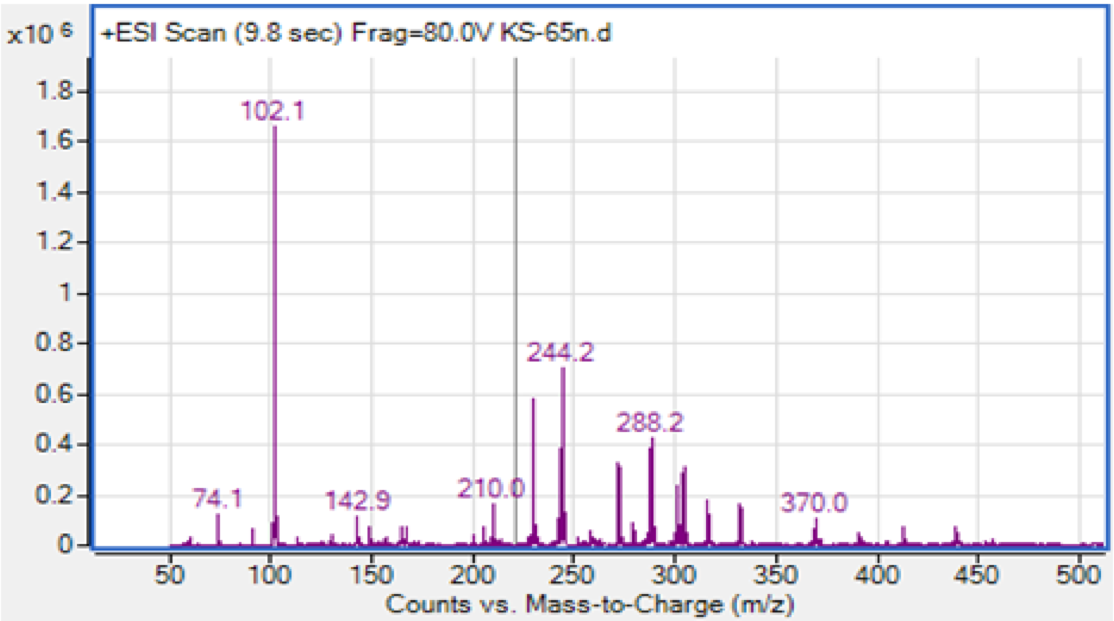

Fig. S9: LC-MS spectrum of **3c**.
